# *APOE* e4 Genotypes Increase Risk of Delirium During COVID-19-Related Hospitalizations: Evidence From a Large UK Cohort

**DOI:** 10.1093/gerona/glab184

**Published:** 2021-06-25

**Authors:** Chia-Ling Kuo, Luke C Pilling, Janice L Atkins, Richard H Fortinsky, George A Kuchel, David Melzer

**Affiliations:** 1 Connecticut Convergence Institute for Translation in Regenerative Engineering, University of Connecticut Health, Farmington, USA; 2 Center on Aging, University of Connecticut Health, Farmington, USA; 3 Epidemiology and Public Health Group, University of Exeter Medical School, UK

Delirium is an acute confusional state and is reported to be particularly frequent and severe in older adults with severe coronavirus disease 2019 (COVID-19) infection (1). Little is currently known of the mechanisms linking COVID-19 to delirium. Given that the *APOE* e4 allele has been associated with delirium (2) and the risk of having severe COVID-19 (3,4) in the general population, we hypothesized that the *APOE* e4 allele may be associated with an increased risk of delirium during COVID-19-related hospitalizations.

To test the hypothesis, we used data from European-ancestry participants of the UK Biobank, recruited at England assessment centers in 2006–2010, with ages between 40 and 70 (*n* = 398 034). The *APOE* genotype was determined based on the genotypes at the single-nucleotide polymorphisms, rs429358 and rs7412, on chromosome 19. Participants with any hospital admission records after the first COVID-19-positive result were included. Delirium cases (ICD-10 codes: F05.0, F05.1, F05.8, F05.9) were restricted to those in the Acute Phase (AP delirium; within 30 days after test-confirmed COVID-19) (5). Those diagnosed after 30 days (long COVID-19) were too few to analyze. Fine and Gray models accounting for the competing risk of mortality were used, adjusting for age, sex, and the top 5 genetic principal components from the European-ancestry participants.

375 173 participants of European descent, visiting England assessment centers at recruitment, were at risk of COVID-19 infection, excluding those who died before the first test date in the data (March 16, 2020, *n* = 22 861). 8725 were tested positive between March 16 and December 31, 2020, of whom those with e3e4 (*n* = 2074), e4e4 (*n* = 251), or e3e3 (*n* = 5098) genotype were further considered. Of the subsample above (*n* = 7423), 51.6% were female and their mean age at the start of the pandemic (set as March 16, 2020) was 65.8 years (*SD* = 8.7). Additionally, 1363 (18.4%) were hospitalized after the first COVID-19-positive result, of whom 958 had COVID-19 diagnosis codes (ICD-10 codes: U071, U072) in their hospital admission records. Five hundred and forty-six died after being tested positive, of whom 414 with causes of death including COVID-19 (mortality data updated to March 2021). Median follow-up time from first COVID-19-positive result to death or last follow-up of hospital admission data (December 31, 2020) was 56 days (range = 0–290 days).

In the e4–delirium association analysis, 1363 participants were hospitalized after test-confirmed COVID-19, of whom 358 were e3e4s (*n* = 49 delirium cases, 43 AP), 64 were e4e4s (*n* = 14, 13 AP), and 941 were e3e3s (*n* = 66, 52 AP). e3e4 and e4e4 genotypes accounted for 47% (56/118) of AP delirium cases from all hospitalized after test-confirmed COVID-19 (*n* = 1575), and 52% (56/108) of AP delirium cases from those restricted to e3e4s, e4e4s, and e3e3s only. Both e3e4s (hazard ratio [HR]_e3e4_ = 2.29, 95% confidence interval [CI]: 1.54–3.41, *p* = 4.80 × 10^–5^) and e4e4s (HR_e4e4_ = 4.94, 95% CI: 2.69–9.09, *p* = 2.80 × 10^–7^) were at higher risk of AP delirium compared to e3e3s. For sensitivity analysis, we used only those with a COVID-19 diagnosis during hospitalization. We also analyzed hospitalized patients excluding those with preexisting dementia before the first COVID-19-positive date and excluding one in third-degree or closer pairs, and the results were consistent in all analyses (Table 1).

**Table 1.** Numbers of Participants With Delirium by *APOE* Genotype and Associations Between *APOE* e4 Genotypes and Delirium in the AP After Test-Confirmed COVID-19

AP Delirium	#e3e3s (#AP delirium, %)	#e3e4s (#AP delirium, %)	#e4e4s (#AP delirium, %)	HR_e3e4_ (95% CI)	HR_e4e4_ (95% CI)
Positive + hospitalized, excluding non-AP delirium (*n* = 1342)	927 (52, 5.6%)	352 (43, 12.2%)	63 (13, 20.6%)	2.29 (1.54–3.41) *p* = 4.80 × 10^–5^	4.94 (2.69–9.09) *p* = 2.80 × 10^–7^
Positive + hospitalized + inpatient COVID-19 diagnosis, excluding non-AP delirium (*n* = 948)	655 (49, 7.5%)	247 (42, 17.0%)	46 (13, 28.3%)	2.43 (1.62–3.64) *p* = 1.80 × 10^–5^	5.13 (2.83–9.30) *p* = 7.0 × 10^–8^
Positive + hospitalized, excluding preexisting dementia and non-AP delirium (*n* = 1236)	877 (43, 4.9%)	309 (29, 9.4%)	50 (7, 14%)	2.07 (1.29–3.30) *p* = .0024	3.74 (1.64–8.52) *p* = .0017
Positive + hospitalized, excluding one in third-degree or closer pairs and non-AP delirium (*n* = 1094)	764 (47, 6.2%)	280 (29, 10.4%)	50 (13, 26.0%)	1.82 (1.15–2.88) *p* = .011	6.47 (3.56–11.75) *p* = 9.0 × 10^–10^

*Notes:* COVID-19 = coronavirus disease 2019; AP = acute phase; HR = hazard ratio; 95% CI = 95% confidence interval. HR comparing e3e4s to e3e3s (HR_e3e4_) and that comparing e4e4s to e3e3s (HR_e4e4_) from Fine and Gray models accounting for the competing risk of mortality, adjusting for age, sex, and the top 5 genetic principal components from the European-ancestry participants in the UK Biobank.

For exploratory purposes, the associations between e4 genotypes and dementia are well established (6) and could possibly hold in patients with test-confirmed COVID-19. Currently, most of the new dementia cases after test-confirmed COVID-19 were recorded in the AP (39 of 55 in e3e4, e4e4, or e3e3 genotypes). While we found that e4 genotypes were associated with increased risk of AP dementia (HR_e3e4_ = 2.24, 95% CI: 1.13–4.44, *p* = .02; HR_e4e4_ = 7.86, 95% CI: 3.06–20.18, *p* = 1.8 × 10^–5^), some of these patients likely developed dementia before COVID-19 infection, which was not captured in the hospital admission records until COVID-19-related hospitalizations. When more post-COVID-19 dementia cases are diagnosed, using additional primary care data would help exclude those with preexisting dementia, and a wash-out period may be set to confirm incident cases.

To conclude, we previously showed that *APOE* e4 genotypes are associated with increased COVID-19 severity (3,4). In this letter, we add that delirium is enriched in e4 genotypes after test-confirmed COVID-19. Inevitably, our study has limitations: included samples restricted to White, hospitalized COVID-19 patients; no proper COVID-19-free controls to compare with for e4–delirium associations; limited data at the pandemic to characterize the samples and to test robustness of the associations. Our findings provide early evidence and need to be confirmed by independent replication in larger samples. Future investigations are needed to understand the underlying mechanisms and to study those not severely infected or hospitalized for long-term health impact from COVID-19 infection.
